# Transcranial random noise stimulation over the primary motor cortex in PD-MCI patients: a crossover, randomized, sham-controlled study

**DOI:** 10.1007/s00702-020-02255-2

**Published:** 2020-09-23

**Authors:** Roberto Monastero, Roberta Baschi, Alessandra Nicoletti, Laura Pilati, Lorenzo Pagano, Calogero Edoardo Cicero, Mario Zappia, Filippo Brighina

**Affiliations:** 1 Department of Biomedicine, Neuroscience and Advanced Diagnostics, University of Palermo, Via La Loggia 1, 90129 Palermo, Italy; 2 Department of Medical and Surgical Sciences and Advanced Technologies “G.F. Ingrassia”, Section of Neurosciences, University of Catania, Catania, Italy

**Keywords:** Parkinson’s disease, Cognitive impairment, Motor cortex, Transcranial random noise stimulation

## Abstract

Mild cognitive impairment (MCI) is a very common non-motor feature of Parkinson’s disease (PD) and the non-amnestic single-domain is the most frequent subtype. Transcranial random noise stimulation (tRNS) is a non-invasive technique, which is capable of enhancing cortical excitability. As the main contributor to voluntary movement control, the primary motor cortex (M1) has been recently reported to be involved in higher cognitive functioning. The aim of this study is to evaluate the effects of tRNS applied over M1 in PD-MCI patients in cognitive and motor tasks. Ten PD-MCI patients, diagnosed according to the Movement Disorder Society, Level II criteria for MCI, underwent active (real) and placebo (sham) tRNS single sessions, at least 1 week apart. Patients underwent cognitive (Digit Span Forward and Backward, Digit Symbol, Visual Search, Letter Fluency, Stroop Test) and motor assessments (Unified Parkinson’s Disease Rating Scale [UPDRS-ME], specific timed trials for bradykinesia, 10-m walk and Timed up and go tests) before and after each session. A significant improvement in motor ability (UPDRS-ME and lateralized scores, ps from 0.049 to 0.003) was observed after real versus sham tRNS. On the contrary, no significant differences were found in other motor tasks and cognitive assessment both after real and sham stimulations. These results confirm that tRNS is a safe and effective tool for improving motor functioning in PD-MCI. Future studies using a multisession tRNS applied over multitargeted brain areas (i.e., dorsolateral prefrontal cortex and M1) are required to clarify the role of tRNS regarding rehabilitative intervention in PD.

## Introduction

Mild cognitive impairment in Parkinson’s Disease (PD-MCI) is rather prevalent in PD, accounting for over 30% of patients (Litvan et al. 2011; Monastero et al. 2018). In 2012 a task force of the Movement Disorder Society (MDS) proposed a standardized set of diagnostic criteria for PD-MCI which improved diagnosis and reduced the heterogeneity of results regarding the determinants and predictors of cognitive impairment in PD (Litvan et al. 2012). Age and disease severity represent the main determinants of PD-MCI (Baiano et al. 2020; Nicoletti et al. 2019). The most frequent PD-MCI phenotype is the non-amnestic (single and multiple domains), and the impairment in executive functioning has been associated with a five-fold increasing risk of PD dementia (PDD) (Litvan et al. 2011; Nicoletti et al. 2019).

The pathophysiological hallmark of PD is a progressive nigro-striatal dopamine deficiency associated with an alteration in cortico-striato-thalamo-cortical circuitry (Wichmann et al. 2011). Transcranial magnetic stimulation (TMS) revealed changes in cortical excitability in PD. Abnormalities in cortico-spinal activation have been documented by studying the motor-evoked potential recruitment at different stimulation intensities (MEP recruitment curve). These studies revealed a greater response at higher intensities with a steeper curve, when compared to the normal, which was observed to correlate with motor impairment, as expressed by bradykinesia and considered to depend upon increased cortical responsivity (Chen and Chen 2019; Udupa and Chen 2019; Bologna et al. 2018). Indeed, the abnormal excitability of intracortical circuits have been found in subjects with PD (even if not in all reports) with evidence of reduced intracortical inhibition and increased intracortical facilitation (Ni et al. 2013; Shirota et al. 2019). Other abnormalities of cortical excitability in PD concerning gender (Kolmancic et al. 2019) and asymmetry of motor involvement have also been reported, particularly in early PD patients, with an interhemispheric imbalance of excitability, and abnormal transcallosal inhibition (Spagnolo et al. 2013).

Furthermore, different cortical areas, which are involved in the execution of a specific movement, are not adequately activated in PD individuals due to an alteration in the thalamocortical inputs (Lefaucheur 2009). Considering the high level of oscillatory synchronization between the cortex and the basal ganglia in PD, repetitive high frequency TMS (HF-rTMS), which was delivered at 5 Hz over the primary motor cortex (M1), was found to induce a dopamine release in the striatal structures (Kim et al. 2008), with a subsequent improvement in the classical PD motor triad (i.e., bradykinesia, rigidity and resting tremor). Accordingly, non-invasive brain stimulation has gained considerable attention in recent years in subjects with PD. TMS and transcranial electric stimulation (TES) are non-invasive techniques, which are capable of modifying neuroplasticity and cortical excitability. Many rTMS studies have demonstrated that repeated stimulation sessions were able to induce lasting therapeutical effects, and that the HF-rTMS approach over M1 is the optimal set, by which to obtain the most effective clinical improvement in PD (Lefaucheur et al. 2004,2020).

To date various studies have evaluated the effect of TES on motor performance in subjects with PD. Fregni et al. (2006) observed that anodal transcranial direct current stimulation (atDCS) applied over M1 improved the Unified Parkinson Disease Rating Scale, Motor Examination (UPDRS-ME) score and simple reaction times in single session study. Conversely, Lu et al. (2018) applied atDCS over the supplementary motor area and they found no effect in self-initiated gait in PD patients with freezing of gait (FOG). When evaluating repetitive session studies, Benninger et al. (2010) revealed a significant improvement of bradykinesia in PD patients, applying atDCS over the motor and prefrontal cortices. And Valentino et al. (2014) evaluated the effect of atDCS over M1 in PD patients, revealing a significant reduction in FOG episodes.

Beyond motor impairment, the dopaminergic fronto-striatal alterations found in PD can lead to a predominant executive dysfunction (Dirnberger and Jahanshahi 2013), which may also occur during the early phases of the disease, thereby causing a reduction in the patient's quality of life (Kehagia et al. 2013). Thus, the early identification of cognitive decline in PD patients can assist in reducing patient’s disability. To date few studies have evaluated the effect of tDCS on cognitive performance in subjects with PD, and these studies used the dorsolateral prefrontal cortex (DLPFC) as a targeting area. Specifically, single session studies demonstrated a significant improvement in working memory, verbal fluency and divided attention in PD subjects (Boggio et al. 2006; Pereira et al. 2013; Bueno et al. 2019); an improvement in executive functioning was also observed in the multiple session study performed by Doruk et al. (2014). Conversely, using a single session atDCS applied over the left DLPFC, Lau et al. (2019) did not report any modification in visual working memory and go/no go performance in PD patients.

Very few studies to date have evaluated the role of tDCS in motor and cognitive functioning in PD-MCI patients. In particular, 2 weeks atDCS over the DLPFC, which was combined with physical therapy in PD-MCI (diagnosed according to Level I MDS criteria) induced an improvement in motor and cognitive abilities (Manenti et al. 2016). In another study, which also applied Level I MDS criteria for PD-MCI, single session atDCS over the medial frontal cortex enhanced theory of mind abilities (Adenzato et al. 2019). Conversely, the only studies which applied Level II MDS criteria for PD-MCI (Biundo et al. 2015; Lawrence et al. 2018), obtained conflicting results: Biundo et al. (2015) did not report a consistent effect on cognitive performance of atDCS applied over the left DLPFC, while Lawrence et al. (2018) observed that cognitive performance improved for those groups receiving cognitive training and tDCS.

Transcranial alternating current and random noise stimulation (tACS and tRNS) has been recently found to modulate specific oscillatory activities, which had been triggered by cognitive tasks in healthy subjects (Antal and Herrmann 2016). Of interest, tRNS applied over M1 was able to induce an increase in cortical excitability with a potentiation of motor-evoked potentials, which were even greater than that produced by atDCS (Terney et al. 2008; Moliadze et al. 2014).

There are very few data in the literature regarding the effect of tACS and/or tRNS in PD patients. Del Felice et al. (2019) recently applied tACS in PD patients and they found a significant improvement in bradykinesia and in a measure of global cognition in patients who had undergone a real versus sham stimulation. Overall, different stimulation settings (e.g., targeting area, single versus repetitive sessions, current intensity, duration of stimulation, etc.) in studies which have evaluated the effect of TES in motor and cognitive performance in PD patients probably account for heterogenous results, in addition to diagnosing MCI in PD (i.e., Level I versus Level II MDS criteria).

M1 encompasses much more than movement organization, and it has been found to be involved in higher cognitive tasks, possessing a functional topography in relation to specific cognitive functional categories (Tomasino and Gremese 2016). Indeed, the involvement of M1 in cognitive processing has been suggested by TMS studies, which showed an effect of motor cortex regarding: executive functioning and working memory, language, visual attention, and semantic memory (Tomasino et al. 2011; Miranda 2013; Lattari et al. 2017; Morya et al. 2019). Recently, a bi-directional change in excitability from the stimulated DLPFC to M1 has been observed in healthy humans (Cao et al. 2018) and rTMS studies have demonstrated that the stimulation of M1 can be modulated by prior stimulation of other cerebral areas, such as the ipsilateral DLPFC (Mastropasqua et al. 2014). Considering the aforementioned evidence, this study tested the hypothesis that tRNS over M1 would induce favourable effects not only on motor performance but also on cognition in PD-MCI patients, who had been diagnosed according to Level II MDS criteria.

## Materials and methods

Patients with PD, who had been diagnosed according to the UK Brain Bank criteria (Hughes et al. 1993) and who belonged to the follow-up cohort of the Parkinson’s Disease Cognitive Impairment Study (PACOS), were enrolled onto the study; PACOS is a multi-center study involving two movement disorders centers in southern Italy (Sicily) (Nicoletti et al. 2019). The aim of the study was to evaluate the frequency of MCI in a large hospital-based cohort of PD patients and its associated clinical features and cognitive biomarkers (Monastero et al. 2018). Specifically, 29 patients, who were consecutively evaluated between January to September 2019 at the Memory and Parkinson’s Disease Center of the “Policlinico Paolo Giaccone” in Palermo, underwent a standard neurological work-up, including: a comprehensive motor examination, and neuropsychological and behavioral assessment described elsewhere (Monastero et al. 2018; Baschi et al. 2018). Inclusion criteria for the current study were: a diagnosis of multidomain PD-MCI according to the MDS level II criteria (Litvan et al. 2012); Hoehn and Yahr stage I–III (Hoehn and Yahr 1967); the use of PD medications at doses which had been stable for at least 8 weeks prior to entering the study; age between 55 and 80 years; at least 5 years of school attendance; and no significant depressive symptoms, according to the Hamilton Depression Rating Scale (HDRS), considering the cut-off score of ≤ 10, as suggested by the MDS (Schrag et al. 2007). Exclusion criteria were: a diagnosis of PD dementia (Emre et al. 2007) and tRNS contraindications (Poreisz et al. 2007). Fourteen (48.3%) out of 29 patients did not meet the inclusion criteria (3 subjects with Hoehn and Yahr stage ≥ 4, 2 with unstable medication, 4 with age ≥ 80 years and/or education < 5 years, and 5 subjects with significant depression). Referring to the exclusion criteria, 3 patients (10.3%) were excluded, 1 for tRNS contraindications and 2 for PD dementia. Furthermore, 2 patients (6.9%) declined to participate in the study, thus leaving 10 patients (34.5%) for the tRNS study. Each patient was examined in the “on” state. All participants provided written informed consent prior to entering the study, which was approved by the local medical Ethics Committee (approval number: 14:03/2018) and in accordance with the Declaration of Helsinki.

### Study design and tRNS protocol

This was a crossover, double-blind, randomized, and sham-controlled study. Both real and sham tRNS were applied in a single session, and performed at least 1 week apart. Patients who underwent real tRNS as an initial treatment were then switched to a sham stimulation, and vice versa. Specifically, counterbalancing was used, in which all of the possible intervention sequences were applied, and all sequences were repeated the same number of times (DePuy and Berger 2005). The patients received an alternating electric current using a battery-powered stimulator (Brainstim stimulator, EMS, Bologna, Italy), which was conveyed through electrodes placed on the scalp. The 4.5 × 4.5 cm electrodes were covered by 7 × 6.5 cm sponges, soaked in saline solution to minimise impedance. According to the 10–20 system, the active electrode was placed over the left M1 and the reference electrode over the contralateral shoulder. Regarding real tRNS, a current of 1.5 mA randomly oscillating in the frequency range of 100–600 Hz was applied for 15 min; in contrast, during sham tRNS the stimulation was turned on for only 30 s (Antal and Herrmann 2016). Fade-in and fade-out phases lasted 10 s for both conditions. Regarding the blinding procedure, the neurologists who administered the treatment and the researchers who analysed the data were both blind to the stimulation condition. Another researcher handled the tRNS device in each treatment session. Concerning the tRNS device, the screen of the machine provides a darkening mode; therefore, neither the patient nor the operator can see the setting parameters.

#### Motor assessment

The evaluation of motor function included: (1) the Italian version of the MDS-UPDRS (Antonini et al. 2013), considering total and lateralized scores (right and left); (2) timed bradykinesia tasks [finger tapping (FT), hand movements (HM), the pronation-supination movements of hands (PS), toe tapping (TT) and leg agility (LA)], evaluating the number of each specific movement executed in ten seconds. The latter were included, because timed tests seem to be more sensitive for detecting changes than UPDRS-ME and they are independent from subjective assessment (Benninger et al. 2010); (3) the Timed Up and Go Test (i.e., the patient is asked to get up from the chair, walk for 3 m, turn around, walk back to the chair and sit down, and the time taken to complete the sequence was calculated) (Bohannon 2006); and (4) the 10-m Walk Test (i.e., the time taken to walk 10 m at a fast pace was timed) (Bohannon 1997).

#### Cognitive assessment

Considering that the non-amnestic (single and multiple domains phenotypes) and that impaired executive functioning and attention are the most frequent cognitive domains and functions affected in PD-MCI (Litvan et al. 2011; Nicoletti et al. 2019), the following cognitive tasks were chosen for analysis: (a) Digit Symbol (DS) (Lezak et al. 2012), which measures visual scanning, sustained attention and visuomotor coordination; (b) Digit Span Forward (DSF) and Backward (DSB) (Monaco et al. 2013), which evaluate attention/short-term verbal memory and working memory, respectively; (c) Visual Search (VS) (Spinnler and Tognoni 1987), which explores selective and divided visual attention; (d) the Stroop Test (ST) (Caffarra et al. 2002), which measures response inhibition, focused attention and executive functioning (ST); and Letter Fluency (LF) (Spinnler and Tognoni 1987), which evaluate frontal/executive functioning. To reduce inter-rater variability, each patient was evaluated by the same neurologist with a specific expertise in neuropsychology.

### Statistical analysis

Statistical analyses were performed using SPSS software (version 21.0 IBM Statistics, IBM Corp). A two-way repeated-measures analysis of variance [with one between-subject factor (sham versus real tRNS) and one within-subject factor (pre versus post-tRNS, T0 and T1)] followed by Duncan post hoc test for multiple comparisons were performed. The significance level was set at *p* < 0.05 for all analyses.

## Results

The demographic and clinical characteristics of PD-MCI are shown in Table 1. In detail, the 10 male PD patients who had been enrolled in the study had a mean age of 70.6 ± 7.8 years, mean age at onset of 65.9 ± 7.4 years, and a mean disease duration of 4.8 ± 4.07 years. All patients were right-handed; the right side of 5 of these patients was affected by the disease, and 5 had the left side affected. The MCI subtype classification was as follows: 4 patients were diagnosed by non-amnestic MCI multi-domain and 6 as amnestic MCI multi-domain. The mean HDRS was 7.5 ± 2.4. Regarding motor assessment, the mean Hoehn and Yahr stage was 1.5 ± 0.5; 5 PD patients had a Tremor Dominant motor phenotype, while 5 had a Postural/Instability and Gait Difficulty phenotype (Stebbins et al. 2013). The Levodopa Daily Dose (LED) was calculated and this sample had a mean LED of 463.9 ± 185.9 mg/daily (Tomlinson et al. 2010).

**Table 1:** Demographic and clinical characteristics of PD-MCI patients

Case	Age (years)	Age at onset (years)	Disease duration (years)	Side affected	MCI type	LED (mg/day)
1	66	64	2	L	naMCI	650
2	59	58	1	R	aMCI	255
3	79	74	5	L	naMCI	300
4	63	60	3	L	aMCI	580
5	78	69	9	L	naMCI	700
6	77	70	7	R	aMCI	400
7	61	55	6	R	aMCI	250
8	68	64	4	L	aMCI	280
9	75	72	3	R	naMCI	660
10	80	79	1	R	aMCI	500

*naMCI* non-amnestic mild cognitive impairment, *aMCI* amnestic mild cognitive impairment, *LED* levodopa equivalent dose *L* left, *R* right

### Motor and cognitive functioning after tRNS

No significant baseline differences have been found for each variables analysed. Subsequently, ANOVA for repeated measures with group and time as factors was used to evaluate the changes caused by tRNS (real versus sham) on motor and cognitive performance. Regarding motor performance, ANOVA revealed a significant main effect of time for the majority of tasks administered (*p* from 0.05 to < 0.0001), probably due to a learning effect. A significant interaction between time × group was found for UPDRS total score (*p* = 0.003), UPDRS lateralized right (*p* = 0.049) and left (*p* = 0.024) scores. Duncan post-hoc comparison showed that UPDRS total score (*p* < 0.0001), UPDRS lateralized right (*p* = 0.001) and left (*p* = 0.004) scores significantly improved only after real tRNS. No interaction between time × group was found for other measures of motor assessment (see Table 2).

**Table 2:** tRNS and motor performances of PD-MCI patients

	tRNS real (mean, SD)		tRNS sham (mean, SD)		*p* value		
	T0	T1	T0	T1	Time	Group	Time × Group
UPDRS-ME tot	32.5 ± 11.7	28.8 ± 12.1	30.9 ± 12.4	30.3 ± 12.7	**< 0.0001**	0.912	**0.003**
UPDRS-ME tot r	11.3 ± 4.8	9.5 ± 4.7	10.2 ± 4.3	9.8 ± 4.5	**0.03**	0.901	**0.049**
UPDRS-ME tot l	12.1 ± 7.5	10.6 ± 6.8	12.6 ± 8.1	12.7 ± 8	**0.05**	0.647	**0.024**
FT r	14.7 ± 6.7	19.3 ± 7.5	18.6 ± 7.4	23.4 ± 5.3	**0.004**	0.161	0.824
FT l	14.4 ± 5.1	21 ± 7.2	21.8 ± 9.4	23.4 ± 11.1	**0.004**	0.159	0.064
HM r	14.5 ± 5.8	18.4 ± 6.2	19.2 ± 5.6	24.1 ± 6.1	** < 0.0001**	0.44	0.646
HM l	14.4 ± 4.7	17.5 ± 5.7	17.4 ± 6.5	21.6 ± 7.6	** < 0.0001**	0.181	0.660
PS r	11.5 ± 2.2	12.3 ± 3.1	12.4 ± 2.9	13.5 ± 4.3	0.066	0.338	0.760
PS l	10.9 ± 3.4	11.7 ± 3.2	12.3 ± 5.4	12.5 ± 4.3	0.321	0.456	0.524
TT r	19 ± 5.7	22.9 ± 7.6	17.5 ± 5.2	19.7 ± 5.2	**0.005**	0.459	0.350
TT l	19.4 ± 9.3	21.7 ± 8.6	17.1 ± 7.3	20.2 ± 8	**0.007**	0.665	0.703
LA r	16.4 ± 5.6	22.5 ± 7.7	17.6 ± 5.1	20.6 ± 7	**0.002**	0.869	0.267
LA l	17.4 ± 7.7	22.7 ± 10.1	17.3 ± 7	19.5 ± 8.6	**0.004**	0.815	0.209
10-m walk (seconds)	6 ± 1	7.4 ± 4.7	5.9 ± 1.6	6.7 ± 3.6	0.256	0.889	0.801
U&G (seconds)	13.6 ± 2.8	13 ± 3.8	12.3 ± 3	11.1 ± 4.7	0.138	0.335	0.704

Bold values are statistically significant

*tRNS* transcranial random noise stimulation, *SD* standard deviation, *UPDRS-ME* Unified Parkinson Disease Rating Scale—Motor Examination, *r* right, *l* left, *FT* finger tapping, *HM* hand movements, *PS* pronation supination, *TT* toe tapping, *LA* leg agility (for all these motor measures the units are no. of movements in 10 s), *U&G* up and go test

Concerning cognitive functioning, a main effect of time was found for Digit Symbol (*p* = 0.019) and Visual Search (*p* < 0.0001). However, no significant interaction between time × group was observed in all the administered cognitive tests after real versus sham stimulation (see Table 3).

**Table 3:** tRNS and cognitive performances of PD-MCI patients

	tRNS real (mean, SD)		tRNS sham (mean, SD)		*p* value		
	T0	T1	T0	T1	Time	Group	Time × Group
Digit symbol	17.1 ± 13.5	20.4 ± 15.6	17.8 ± 10	20.2 ± 13.1	**0.019**	0.810	0.521
Digitspan forward	3.6 ± 0.5	3.4 ± 0.9	3.6 ± 0.5	3.6 ± 0.5	0.500	0.673	0.529
Digitspan backward	2.5 ± 0.7	2.5 ± 0.7	2.2 ± 0.4s	2.2 ± 0.42	0.433	0.224	0.436
Visual search	34.5 ± 10.2	42.1 ± 9.9	37.9 ± 9.1	42 ± 10.4	** < 0.0001**	0.608	0.353
Stroop errors	3.7 ± 5.3	3.9 ± 4.1	2.6 ± 3.52	1.9 ± 3.2	0.722	0.385	0.35
Stroop time	43.1 ± 20.9	40.7 ± 30.2	35.3 ± 28.3	33.5 ± 36.7	0.631	0.393	0.393
Letter fluency	21.4 ± 17.8	21.1 ± 15.1	20.5 ± 14.5	21 ± 13.6	0.961	0.970	0.579

Bold values are statistically significant

*tRNS* transcranialrandom noise stimulation, *SD* standard deviation

## Discussion

The aim of this study was to evaluate the effect of tRNS applied over the left M1 in PD-MCI patients relating to motor and cognitive tasks. The main results were: (1) real tRNS improved motor performance, as demonstrated by reduced UPDRS-ME total and lateralized scores, compared to the sham stimulation. However, real tRNS did not affect other measures of motor function, including timed tasks evaluating bradikynesia and walking ability; and (2) no effect of real versus sham tRNS was observed in cognitive tasks evaluating attention and executive functioning.

Multiple brain regions have been targeted in studies whose aim was to evaluate the effect of neurostimulation in PD patients. PD is a disease with multiple facets, ranging from motor disorders to depressive and cognitive aspects. Specifically, M1 has been targeted when the principal outcome was to induce an improvement in motor tasks (Lee et al. 2019), while studies focusing on cognitive impairment in PD have used DLPFC as a targeted area (Pereira et al. 2013; Doruk et al. 2014). However, recent research suggests the involvement of M1 in cognitive skills including attention, memory, language and executive functioning (Tomasino et al. 2011; Miranda 2013; Lattari et al. 2017; Morya et al. 2019). Furthermore, a bi-directional modulation between DLPFC and M1 has been described by non-invasive brain stimulation studies (Cao et al. 2018; Mastropasqua et al. 2014), thus suggesting that the stimulation of M1 could be involved in cognitive functioning.

Here in particular, tRNS was used because of its ability to induce an enhanced modulation of motor cortical excitability with respect to atDCS. The mechanism underlying such effects remains unclear but it has been suggested that the increased cortical activation by tRNS could rely on the phenomenon of the so-called ‘stochastic resonance’. According to the latter, subthreshold signals, which are too weak to reach activation threshold, can be increased by adding noise. On such a basis, tRNS can act by amplifying sub-threshold neural activities, thus increasing the synchronisation of nervous stimuli (Antal and Herrmann 2016; Paulus 2011).

A recent systematic review concerning non-invasive brain stimulation and motor performance in PD patients has revealed that the atDCS of M1 improved the number and duration of FOG episodes in PD patients with a long-lasting effect (4 weeks after the final session) (Delgado-Alvarado et al. 2020). In agreement with the results of the research presented in this paper, Fregni et al. (2006) have found a beneficial effect of tDCS applied over M1 in improving UPDRS scores and reaction time in PD patients. However, whilst Benninger et al. (2010) demonstrated that atDCS over M1 was able to improve bradykinesia, no difference was found regarding the UPDRS-ME scores. These partly conflicting results are in line with the current literature regarding tDCS in PD, probably reflecting methodological heterogeneity regarding settings (single versus repetitive sessions) and the paradigms used (targeted area, intensity and motor assessment). Different results in tasks evaluating bradykinesia between the research by Benninger’s et al. and that presented in the present research may also be explained by the different method for assessing bradykinesia. Indeed, Benninger’s et al. measured the time to perform each motor sequence ten times, while the present study recorded the number of movements performed in ten second-intervals. Recent research, which has been summarized in a meta-analysis of 18 studies, has suggested that tDCS protocols targeting multiple brain regions (M1 and premotor cortex; M1 and prefrontal cortex; bilateral M1; bilateral DLPFC) may produce improved treatment effects on functional locomotion in PD (Lee et al. 2019). Indeed, FOG does not involve only motor dysfunction, but also executive abnormalities. Accordingly, a very recent tDCS study found that the simultaneous stimulation of M1 and left DLPFC reduced the severity of FOG, thereby suggesting an impaired cortical network between the prefrontal cortex, the motor cortex, and subcortical structures in PD (Dagan et al. 2018).

Cognitive impairment in PD occurs in the early phase of the disease; it is also associated with a worse prognosis in newly-diagnosed subjects (Nicoletti et al. 2019). Previous single session studies, which evaluated the effects of atDCS applied over the DLPFC in PD patients, have revealed a positive effect of stimulation in tasks assessing: working memory, verbal fluency, divided attention and response inhibition (Boggio et al. 2006; Pereira et al. 2013; Bueno et al. 2019). Conversely, Lau et al. (2019) did not report any modification in visual working memory and go/no go performance in PD using a single session atDCS, which was applied over the left DLPFC. Furthermore, repetitive sessions atDCS studies, applied over DLPFC, observed a sustained improvement in divided attention tasks in PD subjects, who received real stimulation compared to the sham group, which persisted after a 1-month follow-up period (Doruk et al. 2014). However, none of the aforementioned studies were either focused on cognitive impairment in PD patients and standardised criteria for PD-MCI were not used.

Using Level I MDS criteria for PD-MCI, Manenti et al. (2016) observed that the combination of cognitive training plus atDCS, which was applied over DLPFC, in PD patients demonstrated an improvement in phonemic verbal fluency performance after 3 months. On the other hand, in Biundo et al. (2015) research, which adopted Level II criteria for PD-MCI, no increase in performance related to immediate memory was observed during the atDCS plus cognitive training treatment (0–4 weeks), and a mere trend to significance appeared at the 16-week follow-up.

To the best of our knowledge, this is the only study which has evaluated the effect of tRNS over M1 on cognitive functioning in PD-MCI subjects, who had been diagnosed according to MDS Level II criteria. No effects of tRNS on executive-attentive performance were observed in this study in PD-MCI patients after stimulating the left M1, and these results confirmed previous research. Indeed, Boggio et al. (2006) have found that atDCS applied over of M1 did not result in a significant improvement in tasks evaluating working memory in patients with PD.

This study has several strengths, including the comprehensive motor assessment, the exclusion of subjects with concomitant depression, and the inclusion of subjects with a diagnosis of PD-MCI, according to the Level II MDS criteria. Furthermore, and to the best of the authors’ knowledge, there are no data in literature regarding the effect of tRNS in PD. However, some limitations should be acknowledged. Firstly, the reduced sample size implied inherent problems of reduced statistical power of the study findings. Secondly, the design of the study: while crossover studies can be characterized by reduced confounding, carry-over effects should be taken into account (Cleophas 2002). Thirdly, only male patients were included in the study, a fact which limits the possibility of generalizing the results. Fourthly, all patients included in this study were classified as multidomain MCI based on their higher risk of conversion to PD dementia, compared to those with single domain (Nicoletti et al. 2019). In a different vein, the subdivision into amnestic versus non-amnestic MCI was not considered due to the nature of the study (i.e., a pilot study*)*. However, a subsequent study with a larger sample have already been planned, to evaluate the role of amnestic versus non-amnestic phenotype in MCI subtypes. Finally, the single session was not probably capable of inducing lasting effects in PD-MCI patients. As final remark, even if a standardized questionnaire which evaluates the occurrence of specific adverse effects and the patient's ability to recognize the type of stimulation received was not used, the blinding was ensured by the fact that the patient did not detect differences during real versus sham stimulation.

## Conclusion

This study confirms that a single session of tRNS over the left M1 is a safe tool, which is capable of inducing modifications in cortical excitability and improving motor function in PD-MCI. On the other hand, the results presented in this paper do not suggest that a single session tRNS over the left M1 is sufficient to improve executive functioning in PD-MCI patients. Different stimulation parameters, including the targeted area, intensity, duration and type of stimulation, may explain the heterogeneity of results described in the present as well as in previous research. Accordingly, further data are required to confirm the role of tRNS in improving cognitive functioning in PD patients. There is also a need for randomised clinical trials regarding the cognitive rehabilitation in PD-MCI patients. To this end, multitarget stimulation, acting on the motor and non-motor symptoms of PD, may represent an effective stimulation modality. Further studies, with a larger sample size and standardized motor and cognitive protocols are warranted. If confirmed, the data may have relevant prognostic and therapeutic implications.
